# Soil chemical factors contributing to differences in bacterial communities among tea field soils and their relationships with tea quality

**DOI:** 10.3389/fpls.2025.1540659

**Published:** 2025-01-30

**Authors:** Shuning Zhang, Naoki Yanagisawa, Mio Asahina, Hiroto Yamashita, Takashi Ikka

**Affiliations:** ^1^ The United Graduate School of Agricultural Science, Gifu University, Gifu, Japan; ^2^ Faculty of Agriculture, Shizuoka University, Shizuoka, Japan; ^3^ Institute for Tea Science, Shizuoka University, Shizuoka, Japan; ^4^ Research Institute of Green Science and Technology, Shizuoka University, Shizuoka, Japan

**Keywords:** soil chemical property, bacterial diversity, bacterial community, tea mineral nutrient, tea quality

## Abstract

**Introduction:**

Soil chemical properties and bacterial communities play key roles in shaping tea plant nutrient status and quality. While the relationships between soil bacterial communities and plant nutrient status have been investigated, the specific role by which soil bacterial communities interacted with soil properties to influence tea plant nutrients and quality remained underexplored.

**Methods:**

In this study, different soil types were collected from tea gardens and designated as soil A (pH 3.41), soil B (pH 3.75), soil C (pH 4.16), soil D (pH 4.17) and soil E (pH 5.56) based on the initial soil pH. We conducted pot cultivation of tea plant ‘Yabukita’ to investigate how soil chemical factors affect bacterial communities and their influences on the nutrient status and quality of tea plants, and finally explored the complex relationships between soil bacterial features and tea quality.

**Results and discussion:**

The results showed that soil bacterial α-diversity was higher level in soils D and E, with distinct β-diversity patterns separating higher pH soils (D and E) from lower pH soils (A, B, and C). The dominant amplicon sequence variants (ASVs) in soils were *Proteobacteria* (28.12%), *Actinobacteriota* (25.65%), *Firmicutes* (9.99%) at phylum level, and *Acidothermaceae* (7.24%), *Solirubrobacteraceae* (4.85%), and *Acetobacteraceae* (4.50%) at family level. Soil pH, exchangeable Mg^2+^, and Ca^2+^ were identified as key factors shaping bacterial community composition and positively correlated with bacterial diversity. Differentially abundant ASVs (DAAs) among all soils were also identified including the phylum *Firmicutes* and families such as *Paenibacillaceae*, *Alicyclobacillaceae*, *JG36-TzT-191*, *KF-JG30-C25*, and *Acidobacteriaceae_subgroup1*. Besides, the nutrient content of tea new leaves varied significantly among soil types and harvests. Combined with Mantel-test association analysis, soil chemical properties and soil bacterial communities were jointly correlated with the contents of total nitrogen, potassium, calcium, aluminum, magnesium, free amino acids, and caffeine in tea new leaves. These findings highlight the dynamic interactions between soil properties, bacterial communities, and tea nutrients, emphasizing the importance of optimizing soil health and bacterial networks to improve tea quality.

## 1 Introduction

Tea [*Camellia sinensis* (L.) O. Kuntze] is an essential crop worldwide, and tea cultivation is a pivotal industry supporting regional development and economies. As a leaf-harvested crop, the nutrient elements and specialized metabolites of tea leaves played a crucial role in tea growth and quality, and maintaining their balance was essential to support sustainable harvesting and pruning practices (Liu et al., 2022; Sun et al., 2019). Among these specialized metabolites, catechins, caffeine, theanine, and aroma compounds were particularly important, which contributed to the rich taste, flavor, and health benefits of tea beverages. Their biosynthesis and accumulation were regulated by complex interactions the genetic background of the tea plants, the environmental conditions, the time of harvest, and the manufacturing process (Li et al., 2022; Fu et al., 2024). In addition, Bag et al. (2022) highlighted the crucial role of the surrounding soil environment, particularly soil nutrient composition and microbial communities, collectively modulating the availability of tea nutrients and influencing metabolic processes in tea plants. Jia et al. (2024) found that soil pH could significantly change the elemental form of the soil and thus affect the uptake and utilization of elements by tea plants. Generally, tea plants were grown in acidic soils, which increased the solubility of aluminum (Al³^+^) and manganese (Mn^2+^) while limiting the activity of certain harmful elements (Yamashita et al., 2020a). Thus, such soils required careful nutrient management, as tea plants depended on soils with balanced nutrient elements and active microbial communities to promote nutrient absorption and quality improvement.

Soil microorganisms, including bacteria, are widely recognized as important bioindicators reflecting the health status of soils and the development and growth of plants, particularly in rhizosphere zone, they can form highly active and complete microbial profiles, participate in nutrient cycling, and respond quickly to changes in the soil environment (Finzi et al., 2015; Hermans et al., 2017). Some studies investigated that the response of bacterial communities to various agricultural management such as rotation management (Soman et al., 2017) and organic fertilization (Hartmann et al., 2015) in many crops. Other studies showed that the effect of soil types on composition structure of bacterial communities was more than that of fertilization (Nicolitch et al., 2016; Li et al., 2019). Besides, soil pH was one of critical factors influencing the composition and activity of soil microbial communities. Chen et al. (2022) indicated that soil bacteria were more sensitive than fungi in response to nutrient inputs, and soil pH could explain the effects of nitrogen enrichment on bacterial communities. In tea soil systems, soil pH was at lower levels due to long-term tea plantation management practices, such as the excessive application of chemical fertilizers and natural acidification processes (Yang et al., 2018). The acidic environment significantly shapes the bacterial community by selecting acid-tolerant bacterial species, which were crucial for nutrient cycling and plant adaptation to such conditions. Chen et al. (2023) investigated that the dominant bacterial taxa in tea plants’ rhizosphere were found to be *Proteobacteria*, *Actinobacteria*, *Chloroflexi*, and *Acidobacteria*, which together accounted for 96% of the bacterial community in the tea rhizosphere. Therefore, further understanding of bacterial composition structure in different soil types plays a crucial role in regulating soil-microbial-plant interactions.

High-throughput sequencing technology, including 16S rRNA sequencing, has become an important research tool for studying soil microbial communities. Ji et al. (2018) and Yi et al. (2022) analyzed detailed information of bacterial communities in tea plantation, their relationships with soil nutrients, and the effects on tea yield. Zhang et al. (2022) found that several beneficial bacteria were of great significance in improving the tea disease resistance and ecological environment during soil bacterial investigation. However, how soil chemical factors affect the composition of bacterial communities and their relationships with tea quality and mineral nutrients remained underexplored. In this study, the pot experiment was conducted with five tea soil types, which to investigate the differences in soil properties, bacterial communities and the nutrient status of tea plants. The aims of our study were: (i) to investigate the relationships among soil properties, bacterial communities, and nutrient status in tea plantations; and (ii) to identify the bacterial diversity and key taxa associated with tea nutrient and quality improvement.

## 2 Materials and methods

### 2.1 Pot culture and experimental design

Tea seedings were transplanted and cultivated inti pots in Wagner pots (capacity: 1/5000 a; one plant per pot) in March 2022. The site was in the greenhouse at Shizuoka University (Shizuoka, Japan). The cultivar was ‘Yabukita’ with a leading tea cultivar in Japan. The experiment utilized five soil types collected from typical tea gardens in Shizuoka, Japan, and analyzed the initial soil properties (**Supplementary Table S1**). To simplify the design of the experiment and minimize the influence of geographical factors such as the location of the tea plantation, the naming of soil types was based on the initial soil pH value, ranked from low to high, and designated as soil A (pH 3.41), soil B (pH 3.75), soil C (pH 4.16), soil D (pH 4.17) and soil E (pH 5.56), respectively. Among these, soil C served as a laboratory conventional soil. Each soil type was represented by two groups: soil with tea plants and soil without tea plants. For each group, five pots per soil type were prepared, constituting five biological replicates. The non-planted soil samples (soil without tea plants) could provide a baseline for comparison to reveal the effects of plant effects. The transplanted tea plants were cultivated under ambient conditions. Daily management such as water, temperature and light exposure were kept consistent across all treatments to minimize environmental variability. Soil moisture was maintained at optimal levels through regular manual watering to prevent water stress or oversaturation. Routine inspections ensured that no weeds or pests interfered with plant growth in the pot. No additional fertilizers were applied during the experiment to focus on the effects of the soil properties and plant effects.

### 2.2 Sampling and analysis of tea plants

After most of tea seedlings had developed five leaves, the new leaves (NL) were sampled in May as the first harvest (1st_NL), and the subsequent new leaves were sampled in July as the second harvest (2nd_NL). Other plant parts, including mature leaves (ML), stems, and roots, were also harvested in July. To determine plant dry matter, all harvested samples were initially weighed to record their fresh weight using an analytical balance. The samples were frozen to preserve their biochemical integrity. Subsequently, the frozen samples were freeze-dried in the lyophilizer for about 48 hours to a constant weight. Once freeze-dried, the samples were re-weighed using a precision analytical balance to determine their dry weight (DW). The freeze-dried samples were then ground into powder for chemical analysis. Plant mineral analyses were performed using inductively coupled plasma-optical emission spectrometry (ICP-OES) (iCAP 7400; Thermo Fisher Scientific, Waltham, USA). The measured minerals included phosphorus (P), potassium (K), calcium (Ca), magnesium (Mg), aluminum (Al), sulfur (S), manganese (Mn), iron (Fe), copper (Cu), boron (B), and sodium (Na). The contents of total N and these minerals were measured by the dry combustion and wet ashing method, respectively. Details of the analytical methods have been described by Yamashita et al. (2020a). The contents of free amino acids (FAAs), catechins, and caffeine were quantified as described by Yamashita et al. (2020b). The total FAAs value represents the sum of nine amino acids: aspartate, asparagine, glutamate, glutamine, serine, arginine, alanine, theanine and γ-aminobutyric acid. The total catechins value represents the sum of seven catechins: (−)-epicatechin, (−)-epicatechin gallate, (−)-epigallocatechin, (−)-epigallocatechin gallate, (+)-gallocatechin, (−)-catechin gallate, and (+)-catechin.

### 2.3 Sampling and analysis of soils

Soil samples were collected in July 2022 from each pot in the experiment. Each tea seedling was carefully removed from its pot, ensuring minimal disturbance to the soil structure. The bulk soil loosely attached to the roots was gently shaken off, leaving only the soil tightly adhering to the root surface. This remaining soil was considered rhizosphere soil. Using a sterilized paintbrush, the rhizosphere soil was meticulously brushed off from the roots into sterile containers. Between samples, all tools were thoroughly cleaned with 70% ethanol to prevent cross-contamination. The rhizosphere soils were immediately separated into two parts. One part was stored in sterilized, pre-labeled tubes at −80°C for subsequent DNA extraction and bacterial community analysis. The other part was air-dried at room temperature (22–25°C) in a clean and ventilated environment, with regular mixing to ensure uniform drying. Once dried, the soils were passed through a sterilized 2-mm sieve to remove debris and roots and stored in containers for the analysis of soil chemical properties. For soils from pots without tea plants, the same sampling protocol was followed. Bulk soil was first gently shaken, and the remaining tightly adhered soil was collected with a sterilized paintbrush to maintain consistency. All samples were processed under the same conditions to minimize variability. The soil pH (soil/H_2_O=1:2.5, w/v) was measured with the pH meter (LAQUA F-74, Horiba, Japan). Soil total carbon (TC) and total nitrogen (TN) contents were determined using the Vario MAX cube analyzer (Elementar, Hanau, Germany) with aspartic acid as the standard. Soil total phosphorus (P) was determined following the wet ashing method and analyzed using the vanadomolybdate method. The reagents used included 0.25% ammonium metavanadate, 5% ammonium molybdate, and 5 N nitric acid solutions, and absorbance was measured at 440 nm with a phosphoric acid standard solution as the reference. Soil exchangeable K^+^, Ca^2+^, Mg^2+^, and Mn^2+^ were extracted using 1 M ammonium acetate (pH 7.0) and quantified using inductively coupled plasma–optical emission spectrometry (ICP-OES; iCAP 7400; Thermo Fisher Scientific).

### 2.4 DNA extraction, amplification, and purification

Genomic DNA was extracted from 0.25 g soil using the DNeasy PowerSoil Pro Kit (Qiagen, Hilden, Germany), according to the manufacturer’s instructions. The quality and quantity of the resulting DNA were examined using a Qubit^®^ 2.0 Fluorometer (Thermo Fisher Scientific). The V4 region of the bacterial 16S rRNA gene was amplified by PCR using the general bacterial primers: 805R (5’-GGACTACHVGGGTWTCTAAT-3’) and 515F (5’- GTGCCAGCMGCCGCGGTAA-3’). The amplification reaction was performed with Tks Gflex™ DNA Polymerase (Takara, Otsu, Japan) under the following thermal cycling conditions: 94°C for 1 min for initial denaturation, followed by 28 cycles of 10 s at 98°C for denaturation, 15 s at 50°C for annealing, and 15 s at 68°C for extension, with final extension at 68°C for 5 min. The PCR products were further purified using magnetic speed beads based on Solid Phase Reversible Immobilization technology (Rohland and Reich, 2012). Then, the PCR products of the 16S rRNA regions were subjected to an additional PCR step to link Illumina sequencing adaptors and sample identifier indexes to the amplicons. The thermal cycling program for this PCR step was as follows: 10 cycles at 98°C for 10 s, 60°C for 15 s, and 68°C for 5 s, and final extension at 68°C for 5 min. The products were then purified using the same method as in the previous step. To prepare a dual-indexed library, index 1 (i7) and index 2 (i5) sequences were added to the amplicons to generate unique targets. Full information for index sequences is provided in **Supplementary Table S2**.

### 2.5 16S rRNA sequencing and data processing

The dual-indexed library was sequenced on the Illumina NovaSeq 6000 platform (paired-end 250-bp) at Novogene Japan. The raw sequencing data were processed using Quantitative Insights into Microbial Ecology (QIIME2) software (https://qiime2.org/). The raw sequencing data in Fastq files were imported and filtered to discard ambiguous nucleotides, low-quality reads, and short-length reads, and then the quality scores of forward and reverse reads were checked (https://view.qiime2.org/). In this study, amplicon sequence variants (ASVs) were obtained instead of Operational Taxonomic Units as outputs of the DADA2 plugin, and the individual elements of the ASVs are referred to as features. The sequences were clustered with 100% similarity after error correction and removal of sequence errors (denoising) to infer sequence variants at single-nucleotide resolution. Taxonomic identification of bacteria was conducted using the feature-classifier plug-in and the Silva 138 reference alignment, using the 515F/806R region of sequences (silva-138-99-515-806-nb-classifier.qza). The α-diversity of bacterial communities was characterized based on Chao1 and Shannon’s indexes, which were calculated from the observed ASVs. To verify the number of reads required for α-diversity analysis, rarefaction curve analysis was used to determine the appropriate read depth. As shown in **Supplementary Figure S1**, for each soil sample, the curve was close to flat, indicating that the observed ASVs and Shannon’s index increased and finally reached saturation as the number of reads increased (**Supplementray Figure S1**). Principal coordinate analysis (PCoA) was conducted to illustrate β-diversity, i.e., the overall differences in bacterial community composition based on Weighted Unifrac distance. Finally, Qiime2 output files (.qza) were imported into R software (version 4.2.1) to visualize the results of bacterial community analyses using the R package qiime2R (ver. 0.99.6).

### 2.6 Statistical analyses

Significant differences in the mineral contents of tea leaves among the five soil types were determined using one-way analysis of variance (ANOVA), while differences in soil properties between soils with plants and soils without plants, as well as among the five soil types, were analyzed using two-way ANOVA. *Post-hoc* analyses were conducted using Tukey’s tests, and differences were considered statistically significant at *p* < 0.05. The Venn diagrams illustrating bacterial communities in different soils were constructed using the R package “ggvenn” (ver. 0.1.9). Bacterial diversity analyses were conducted to assess the α- diversity and β-diversity using “tidyverse” (ver. 2.0.0). The soil DAAs were identified using the “topTags” function from the R package “edgeR” (ver. 3.40.2), based on log fold change (logFC), P-value, and False Discovery Rate (FDR). The correlations between important bacterial traits and environmental variables were assessed using the Mantel test with the R package “linkET” (ver. 0.0.7.4).

## 3 Results

### 3.1 The soil chemical properties influenced by soil types and tea plants

We analyzed each of the five soils after cultivation with and without tea plants to determine the key chemical properties (**Table 1**). The results showed that the soil pH was lowest in soil A with plants (3.31) and highest in soil E with plants (6.37), and the pH value was significantly influenced by the plant effects. The EC value was higher in soils A, B and E (without plants) than that in soil with plants, which was also significantly influenced by the plant effects. The total P and N contents of soil B and D was significantly higher than that of other soils. The C/N ration of soil D was significantly lower than that of other soils. The exchangeable Mg^2+^, and Ca^2+^ contents of soil D and E were significantly higher than that of other soils, which were also influenced by the plant effects. Since soil C was set to the conventional soil, the soil EC value, total P, total N, total C, and exchangeable cations contents were much lower than in the other soils. In addition, based on the results of the two-way ANOVA, all analyzed soil properties were significantly affected by soil type. Soil pH, EC, total P, exchangeable K^+^ and Ca^2+^ were significantly affected by plant effects. The interaction between soil type and the presence of tea plants was significant for soil pH, EC value, and exchangeable cations (Mg^2+^, Ca^2+^,K^+^ and Mn^2+^).

**Table 1:** The soil properties in different soil types.

Soil type	pH (H_2_O)	EC (ms·cm^-1^)	Total P (g·kg^-1^)	Total N (g·kg^-1^)	Total C (g·kg^-1^)	C/N	Exchangeable-Ca^2+^ (g·kg^-1^)	Exchangeable-K^+^ (g·kg^-1^)	Exchangeable-Mg^2+^ (g·kg^-1^)	Exchangeable-Mn^2+^ (g·kg^-1^)
A with plants	3.31 ± 0.14f	0.23 ± 0.04cd	10.26 ± 0.67d	37.00 ± 1.09a	431.96 ± 9.47a	11.70 ± 0.58a	0.73 ± 0.02e	0.72 ± 0.03c	0.29 ± 0.02de	0.07 ± 0.00a
A without plants	3.48 ± 0.06f	0.34 ± 0.04a	11.10 ± 0.76cd	36.60 ± 1.00a	418.70 ± 7.62a	11.63 ± 0.26a	0.55 ± 0.043e	0.75 ± 0.04c	0.20 ± 0.02e	0.05 ± 0.00b
B with plants	3.73 ± 0.10e	0.15 ± 0.02e	12.60 ± 0.35b	25.84 ± 2.01b	289.72 ± 7.91bc	11.26 ± 0.71a	1.56 ± 0.11d	0.47 ± 0.05e	0.21 ± 0.03e	0.02 ± 0.00d
B without plants	3.77 ± 0.11de	0.16 ± 0.02e	12.75 ± 0.43b	26.98 ± 2.22b	297.28 ± 3.42b	11.08 ± 0.83ab	1.90 ± 0.02d	0.92 ± 0.08b	0.34 ± 0.08d	0.04 ± 0.01c
C with plants	3.97 ± 0.03cd	0.06 ± 0.00f	6.43 ± 0.27e	6.06 ± 0.74d	68.74 ± 8.20f	11.40 ± 1.18a	0.10 ± 0.01f	0.23 ± 0.03f	0.05 ± 0.01f	0.02 ± 0.00d
C without plants	4.10 ± 0.06c	0.07 ± 0.00f	6.13 ± 0.41e	5.24 ± 0.57d	56.18 ± 2.73f	10.78 ± 0.94ab	0.10 ± 0.01f	0.27 ± 0.03f	0.05 ± 0.01f	0.02 ± 0.00d
D with plants	4.72 ± 0.12b	0.18 ± 0.03de	17.58 ± 0.72a	33.52 ± 2.32a	265.11 ± 16.75d	7.87 ± 0.23c	3.09 ± 0.12c	0.98 ± 0.01b	0.94 ± 0.07c	0.07 ± 0.01a
D without plants	4.59 ± 0.12b	0.33 ± 0.06ab	18.70 ± 0.79a	34.22 ± 2.67a	268.50 ± 19.74cd	7.86 ± 0.21c	3.00 ± 0.10c	1.44 ± 0.01a	1.00 ± 0.05c	0.07 ± 0.01a
E with plants	6.37 ± 0.05a	0.27 ± 0.03bc	11.18 ± 0.70cd	14.45 ± 1.03c	146.58 ± 6.49e	10.19 ± 1.04ab	9.19 ± 0.27a	0.55 ± 0.02de	1.52 ± 0.02a	0.005 ± 0.00e
E without plants	6.49 ± 0.14a	0.33 ± 0.02ab	11.73 ± 0.63bc	14.36 ± 1.15c	138.60 ± 2.81e	9.69 ± 0.62b	8.20 ± 0.33b	0.62 ± 0.03cd	1.37 ± 0.06b	0.004 ± 0.00e
Statistical Analysis										
Soil type	*p* < 0.001	*p* < 0.001	*p* < 0.001	*p* < 0.001	*p* < 0.001	*p* < 0.01	*p* < 0.001	*p* < 0.001	*p* < 0.001	*p* < 0.001
Plant effect	*p* < 0.05	*p* < 0.001	*p* < 0.01	NS	NS	NS	*p* < 0.01	*p* < 0.01	NS	NS
Interaction	*p* < 0.05	*p* < 0.001	NS	NS	NS	NS	*p* < 0.001	*p* < 0.001	*p* < 0.001	*p* < 0.001

Values are mean ± SD (n = 5). Different letters indicate that means are significantly different (Turkey’s HSD test); Significant differences among five soil types of with plant or without plant effects were estimated by two-way ANOVA. NS, No Significant.

### 3.2 Soil bacterial communities and their relationships with soil properties

**Figure 1:**
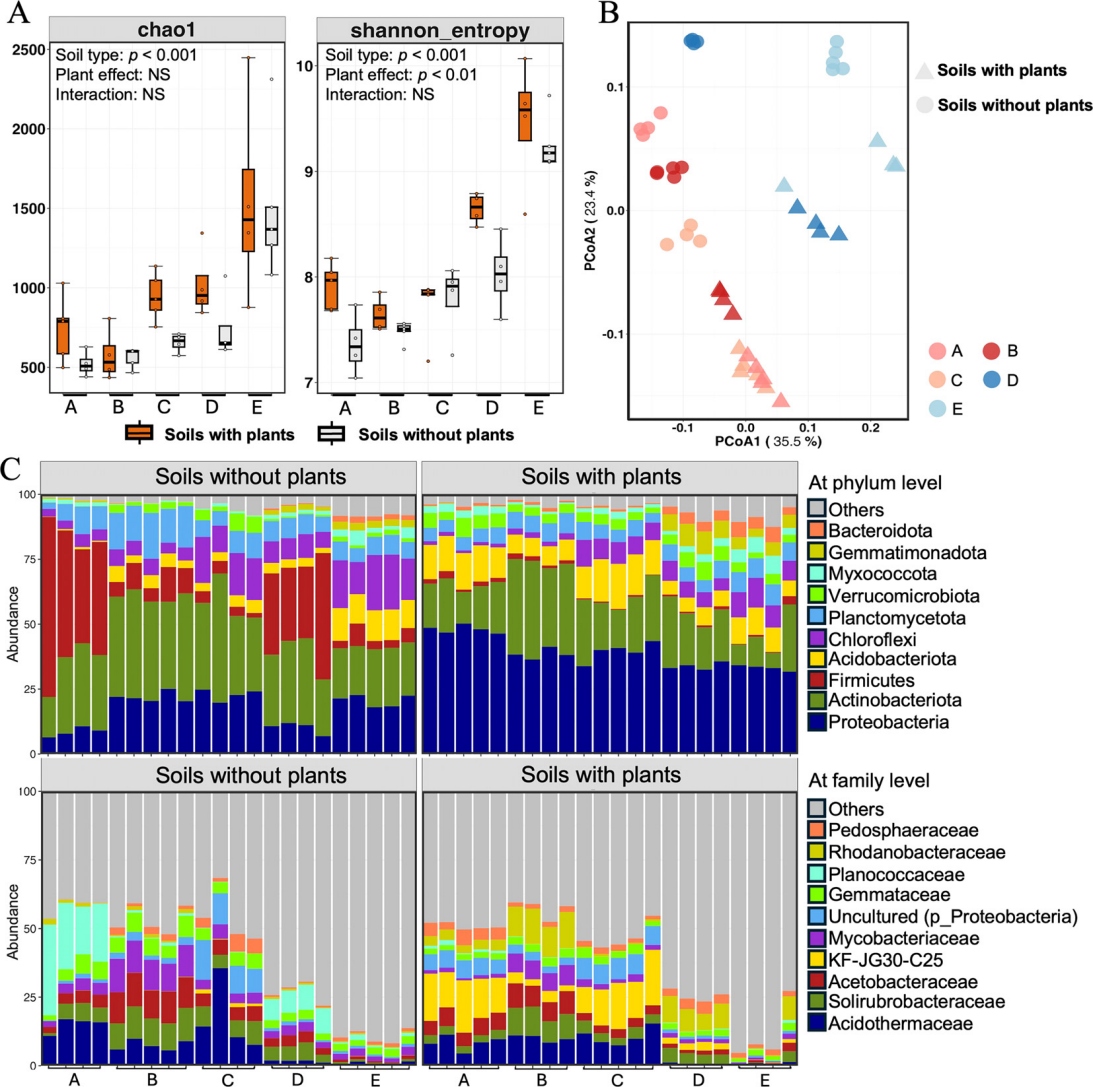
The diversity and composition of bacterial communities in different soil types. **(A)** The alpha-diversity (Chao1 and Shannon index) of soil bacterial communities. **(B)** The PCoA analysis of soil bacterial communities. **(C)** The relative abundances of top 10 bacterial communities at the phylum (upper) and family (lower) levels from different soils. Letter A, B, C, D and E refer to soil A, soil B, soil C, soil D and soil E, respectively.

A total of 2,403,668 high-quality sequences were obtained with a median read count per sample of 54,629. On average, 89.93% of the original sequences passed the quality filter, and 86.77% of the denoised sequences were non-chimeric, resulting in an overall effective sequence recovery rate of 79.52% (**Supplementary Table S3**). Firstly, we analyzed the α-diversity by calculating Chao1 and Shannon’s indexes to quantify the diversity and richness of soil bacterial communities (**Figure 1A**). Significant differences in α-diversity were detected among the different soil types. The Chao1 index was highest for soil E with plants, followed by soil D, and lowest for soil B. The Chao1 index was slightly higher for soils with plants than for soils without plants, but the differences were not significant. The Shannon’s index showed a similar trend. The PCoA analysis based on the weighted-unifrac distance showed the β-diversity of soil bacterial communities (**Figure 1B**). The first principal coordination axis accounted for 35.5% of the variation in soil bacterial communities, and the second principal coordination axis explained 23.4%. The results showed there was a clear separation of soil types between bule set (soil D and E) and red set (soil A, B, and C), and there were also obvious differences between soil with plants and soil without plants. After feature classification, a total of 11,620 ASVs were retained across all soil samples. The dominant ASVs at the phylum and family levels in different soil types are summarized in **Figure 1C**. The dominant phyla were *Proteobacteria* (28.12%), *Actinobacteriota* (25.65%), *Firmicutes* (9.99%), *Acidobacteriota* (7.78%), *Chloroflexi* (7.78%), and *Planctomycetota* (7.52%), which together accounted for the large proportions of the bacterial communities. The dominant families were *Acidothermaceae* (7.24%), *Solirubrobacteraceae* (4.85%), *Acetobacteraceae* (4.50%), *KF-JG30-C25* (4.03%), and *Mycobacteriaceae* (3.74%).

**Figure 2:**
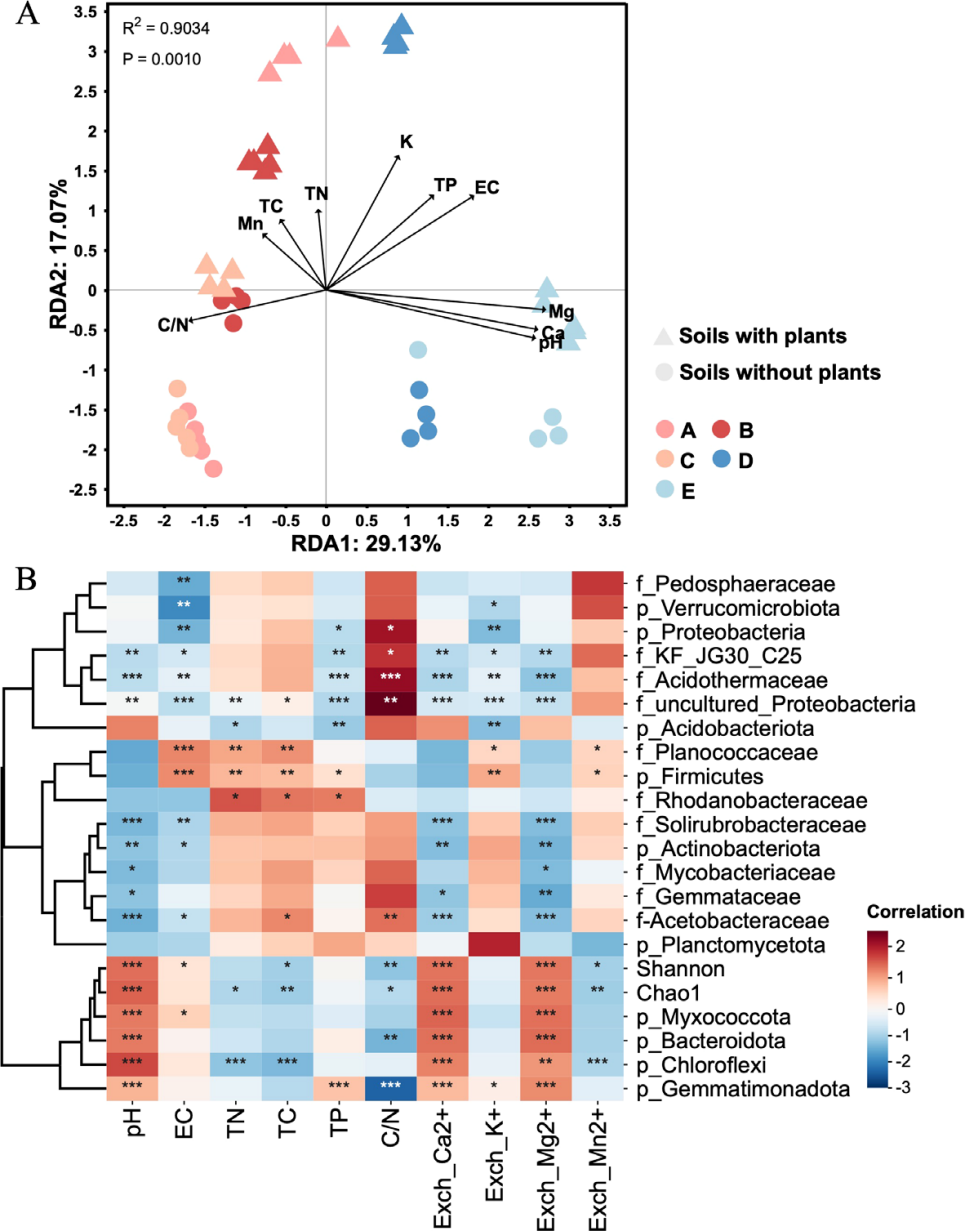
Relationship between soil chemical properties and bacterial communities. **(A)** The redundancy analysis (RDA) between soil chemical properties and bacterial composition across different soil types. Letter A, B, C, D and E refer to soil A, soil B, soil C, soil D and soil E, respectively. **(B)** The correlation analysis between soil chemical properties and dominant bacterial ASVs (top10 phyla and top 10 families) and bacterial diversity (Chao1 and Shannon). * *p* < 0.05, ** *p* < 0.01, and *** *p* < 0.001. Red and blue color indicate positive and negative correlations with Pearson’s correlation analysis.

The redundancy analysis revealed correlations between soil chemical properties and bacterial community composition across different soil types (**Figure 2A**). The results showed that soil pH exerted a strong influence on the composition and structure of bacterial communities, followed by exchangeable Ca^2+^ and Mg^2+^. Other factors, such as exchangeable K^+^, EC, and C/N, contributed to variations in bacterial composition, albeit to a lesser extent. In addition, the correlation analysis was conducted to visually quantify relationships between soil chemical properties and dominant bacterial ASVs (**Figure 2B**). The results showed the soil pH, exchangeable Ca^2+^ and Mg^2+^ were positively correlated with bacterial diversity (Chao1 and Shannon) and the abundance of *Gemmatimonadota*, *Myxococcota*, *Bacteroidota*, and *Chloroflexi*, and negatively correlated with the abundance of *Acidothermaceae*, *Solirubrobacteraceae*, and *Acetobacteraceae*. The contents of total C,N and P were positively correlated with the abundance of *Firmicutes* and *Rhodanobacteraceae*. The C/N value was positively correlated with the abundance of *Proteobacteria*, *KF-JG30-C25*, and *Acidothermaceae*, and negatively correlated with bacterial diversity and the abundance of *Bacteroidota* and *Gemmatimonadot*a. Thus, soil pH was the most important factor affecting the soil bacterial communities, and other chemical factors, such as Ca^2+^ and Mg^2+^, had a significant impact on the distribution of bacterial community.

### 3.3 Key bacteria with significant abundance and functional prediction

**Figure 3:**
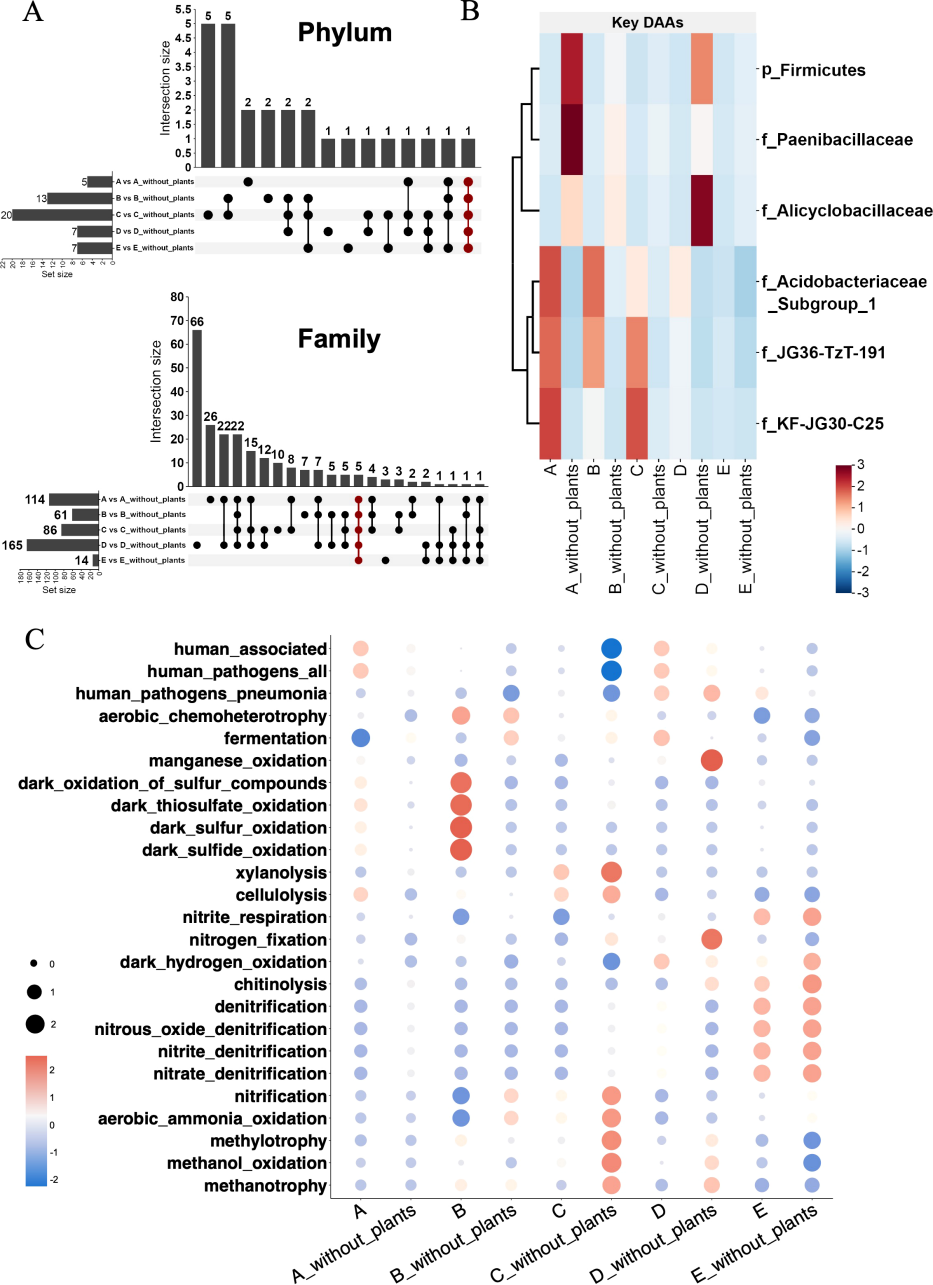
The key bacteria with significant abundance and functional prediction. **(A)** The upset diagram of unique ASVs in different soil types at phylum and family level. **(B)** The heatmap of key bacteria with significant abundance. **(C)** The FAPROTAX diagram of bacterial function prediction. Letter A, B, C, D and E refer to soil A, soil B, soil C, soil D and soil E, respectively.

Compared to soil without plants, unique ASVs in soil with plant could provide special functions and confer adaptability to plant development and the common ASVs in all soils reflected the dominant effect of soil environments. To identify differentially abundant ASVs (DAAs) associated with plant effects, we compared ASV abundances between soils with plants and their corresponding soils without plants at the phylum and family levels (**Figure 3**). At phylum level, the numbers of DAAs in each soil set were A vs A_without_plants (5), B vs B_without_plants (13), C vs C _without_plants (20), D vs D_without_plants (7), and E vs E_without_plants (7). At family level, the numbers of DAAs in each soil set were A vs A_without_plants (114), B vs B_without_plants (61), C vs C _without_plants (86), D vs D_without_plants (165), and E vs E_without_plants (14). Common DAAs across all soil sets were also identified (red points in **Figure 3A**), representing microbial taxa that were consistently enriched or depleted regardless of different soil types. These included representatives of the phylum Firmicutes and families such as *Paenibacillaceae*, *Alicyclobacillaceae*, *JG36-TzT-191*, *KF-JG30-C25*, and *Acidobacteriaceae_subgroup1* (**Figure 3B**).

Functional Annotation of Prokaryotic Taxa (FAPROTAX) was applied to quickly identify and interpret the functional differences of bacterial communities in different soil types (**Figure 3C**). The results showed that a variety of functions in soil bacterial community were involved, including the nitrogen cycle (nitrification, denitrification and nitrogen fixation), organic decomposition (cellulose decomposition and xylanolysis), sulfur cycle (dark sulfide and sulfur oxidation, thiosulfate oxidation), and other functions. In soils with plants (A_with_plants, B_with_plants), the abundances of functions including cellulose decomposition and sulfur cycle increased significantly. In soils with plants (D_with_plants, E_with_plants), nitrite denitrification, nitrate denitrification, and nitrous oxide denitrification, as the essential part of the denitrification pathway, were core ecosystem functions in soil bacterial communities. Besides, more aerobic methanotroph activity was supported in soil without plant (B_without_plants, C_without_plants and D_without_plants).

### 3.4 The nutrients status of tea plants and their relationships with soil properties and bacterial community

**Figure 4:**
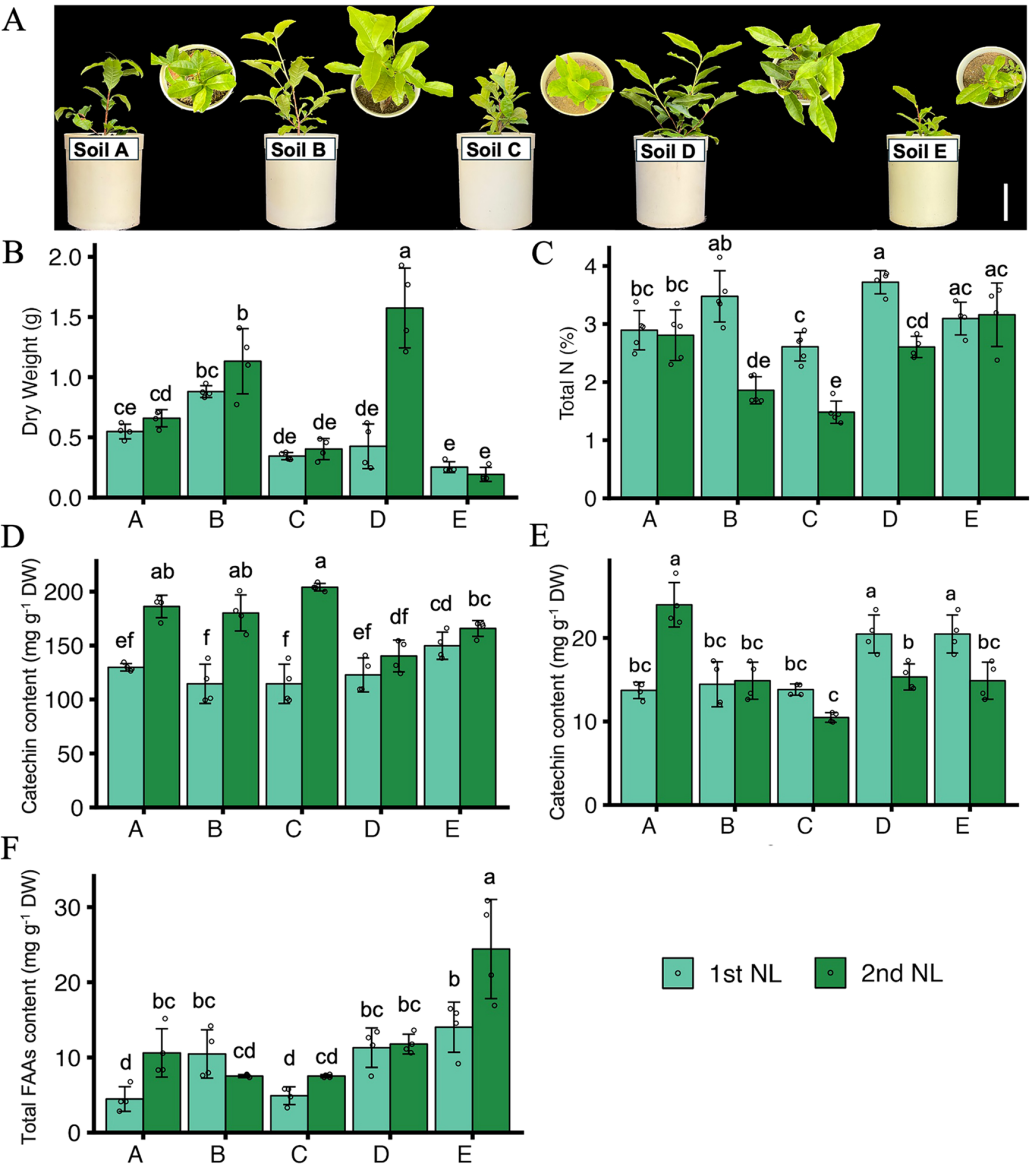
Growth condition and quality indexes of tea plants. **(A)** Growth condition of tea plants in pots. Bar = 10 cm. **(B)** Dry weight (DW) of new tea leaves. **(C)** Total N contents in new tea leaves. **(D)** Total catechin contents in new tea leaves. **(E)** Total caffeine contents in new tea leaves. **(F)** Total free amino acids content (FAAs) in tea new leaves. 1st NL: new leaves in the first harvest; 2nd NL: new leaves in the second harvest. Letter A, B, C, D and E refer to soil A, soil B, soil C, soil D and soil E, respectively. Values and error bars are mean ± SD (n = 5). Different letters above bars indicate significant differences in each panel (Tukey’s HSD test, *p* < 0.05).

**Figure 5:**
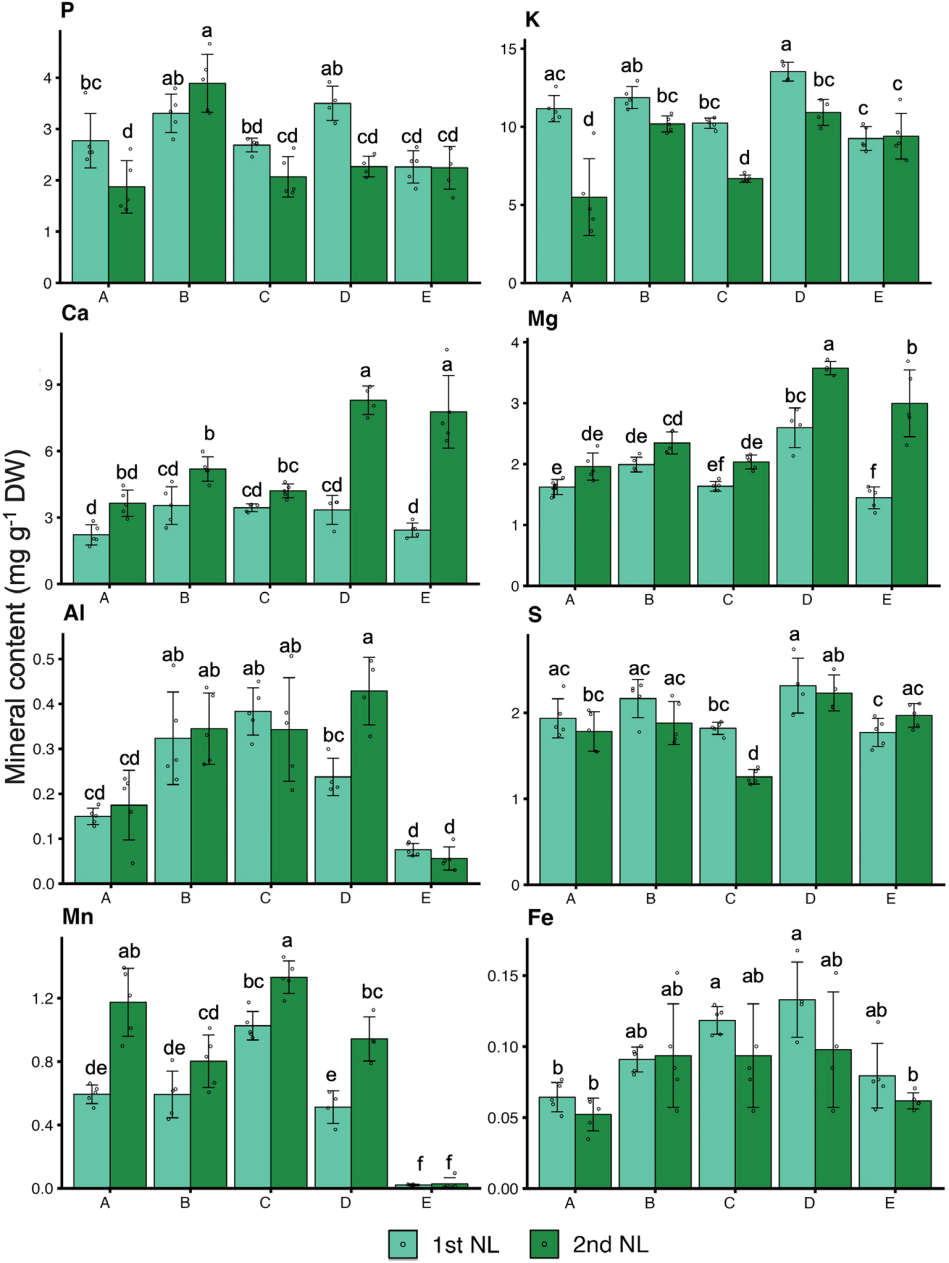
Mineral contents in tea new leaves. Values and error bars are mean ± SD (n = 5). Different letters above bars indicate significant differences in each panel (Tukey’s HSD test, *p* < 0.05). Letter A, B, C, D and E refer to soil A, soil B, soil C, soil D and soil E, respectively. 1st_NL: new leaves in the first harvest, 2nd_NL: new leaves in the second harvest.

To comprehensively evaluate the growth and nutrient status of tea plants, we investigated the growth, quality indexes, and mineral contents of NL, ML, and roots (**Figures 4**, **5**; **Supplementary Table S4**). The growth condition of tea plants differed among the different soil types (**Figure 4A**). The dry weight of the NL at the second harvest was higher in plants grown in soil B and soil D than in plants grown in the other soils, and the DW of the NL at both harvests was significantly lower in plants grown in soil E than in those grown in the other soils (**Figure 4B**). The total N contents of the NL at the first harvest were significantly higher in plants grown in soil B and soil D than in those grown in the other soils (**Figure 4C**). In the NL at the first harvest, the catechin content did not differ significantly among the plants in the five soil types, but the caffeine contents were lower in plants grown in soils A, B, and C than in those grown in soils D and E. In the NL at the second harvest, the catechin contents were higher in plants grown in soils A, B, and C than in those grown in soils D and E, and the caffeine content was significantly higher in plants grown in soil A than in those grown in the other soil types (**Figures 4D, E**). The FAAs content was highest in plants grown in soil E, especially in the second harvest, followed by those grown in soil B and D (**Figure 4F**). Besides, the contents of macronutrients and some micronutrients in tea leaves and roots were determined (**Figure 5**; **Supplementary Table S4**). The P, K, S, and Fe contents in the NL at the first harvest were significantly higher in plants grown in soils B and D than in those grown in soils A, C, and E. The Ca and Mg contents in the NL at the second harvest were significantly higher in plants grown in soils D and E than in those grown in soils A, B, and C (**Figure 5**). Compared with the plants grown in soils A, B, D, and E, those grown in soil C had significantly higher contents of Cu, B, Fe, and Al, and lower contents of S and Mg in the ML (**Supplementary Table S4**). In the roots, the contents of P, K, Ca, and Mg were lower in plants grown in soil A than in those grown in the other soils, and the contents of Fe, S, Ca, and Mg were higher in the plants grown in soil E than in those grown in the other soils (**Supplementary Table S4**).

**Figure 6:**
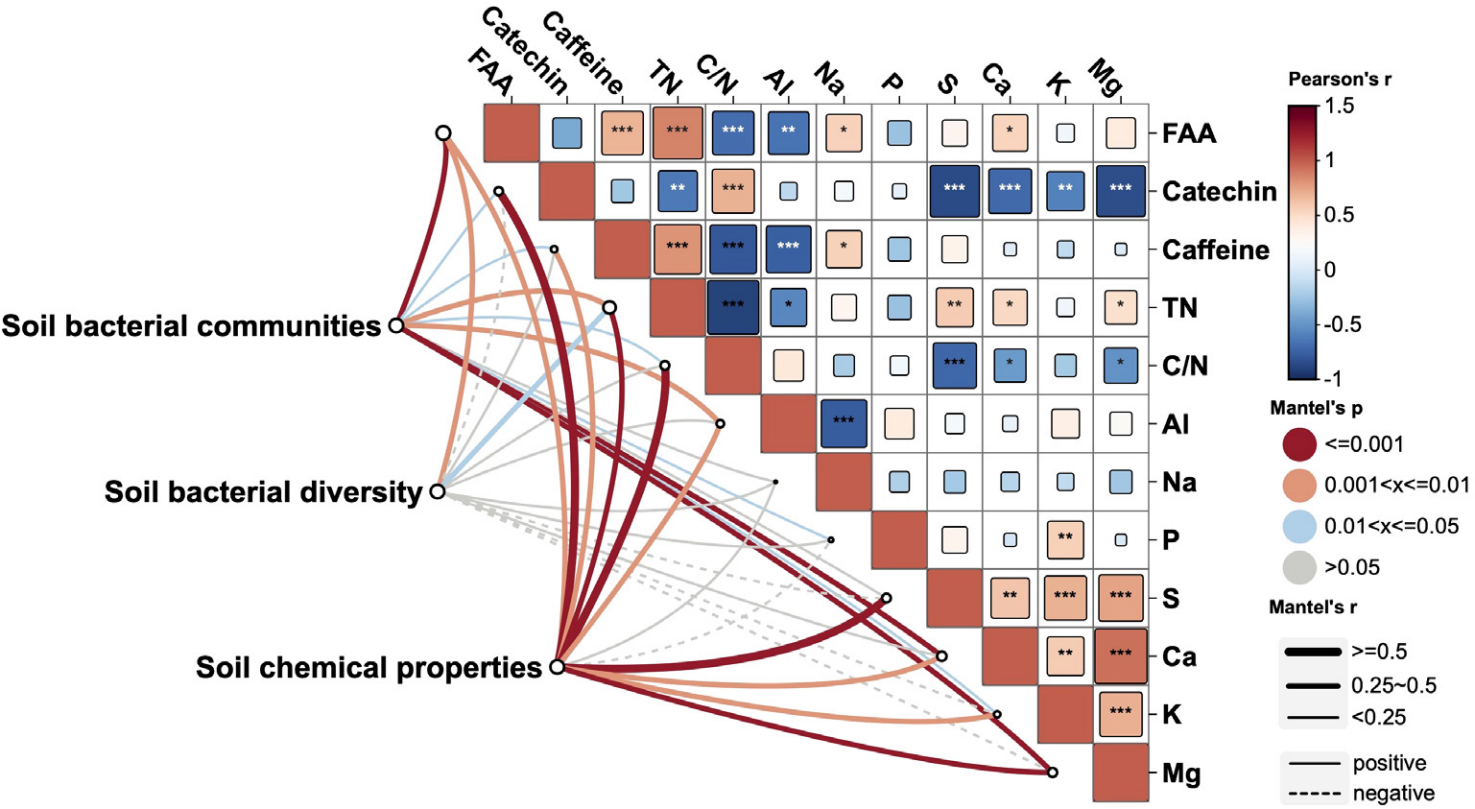
Relationships between soil features (bacterial communities, bacterial diversity and soil chemical properties) and tea nutrient status based on Mantel’s test. Tea nutrient status of tea new leaves at the second harvest: phosphorus (P), potassium (K), calcium (Ca), magnesium (Mg), aluminum (Al), sulfur (S), sodium (Na), free amino acid (FAA), and total nitrogen (TN). *, **, and *** indicate significant differences at p < 0.05, p < 0.01 and p < 0.001.

Mantel tests were performed to further evaluate the relationships between bacterial features (bacterial diversity and communities) and nutrient status (mineral and quality contents) of tea new leaves (**Figure 6**). The FAAs content was positively related to the contents of TN, Caffeine, Ca and Na in tea new leaves, and was negatively related to the C/N ration and Al contents. The catechin content was positively related to the C/N ration, and was negatively related to the contents of TN, P, S, K and Mg. Besides, soil chemical properties, soil bacterial communities and bacterial diversity were jointly correlated with the contents of FAAs and TN. Soil bacterial communities and soil chemical properties were jointly correlated with the contents of catechins, caffeine, C/N ration, Al, Ca, K and Mg. Soil bacterial communities was correlated with the contents of P, and soil chemical properties was correlated with the contents of S (**Figure 6**).

## 4 Discussion

Microorganisms are being increasingly recognized as an indispensable part of the soil ecosystem in tea plantations. This ecosystem plays an important role in the growth and development of tea plants. In this study, we investigated the chemical properties and bacterial communities in soils from different tea fields, and determined how various soil factors contribute to tea quality.

### 4.1 Effects of different soil types on soil properties and bacterial diversity

In this study, soil with lower pH (A and C) showed limitations in cation availability (Ca^2+^, Mg^2+^) despite higher total N and C contents, while soil with higher pH (D and E) could provide a more balanced nutrient profile conducive to plant growth (**Table 1**). Soil pH has been recognized as an important factor influencing soil biological and physicochemical processes (Neina, 2019). For example, soil cations (Ca^2+^, Mg^2+^, Na^+^) decreased with the soil pH reduction because of H^+^ occupied many exchange sites and crowded out the positions of other cations, finally leading to leaching (Meng et al., 2019). Besides, the plant effects primarily affected pH, EC and exchangeable cations, which reflected active nutrient uptake processes (**Table 1**). Organic acids, fatty acids and some signalling molecules exuded by tea root could affect the surrounding soil environment and regulate the absorption of plant nutrients (Jiang et al., 2024), which is the reason for the difference between soil with plant and without plant. However, this effect on total N, C and P contents were minimal, suggesting that the short experimental period was insufficient for significant plant-induced depletion of these nutrients. These findings emphasize the need for tailored soil management strategies to optimize nutrient availability and improve tea plant productivity based on specific soil types. However, this study only collected soil samples after the harvest period of tea new leaves, which limited tracking changes in soil properties with plant growth. In future studies, non-destructive soil sampling methods or increasing sample sizes could be explored to enable more frequent soil data collection without impacting the experimental conditions. This would provide a more dynamic result about soil properties changes throughout the plant growth cycle.

Soils exhibit heterogeneity and diversity in terms of their physical, chemical, and biological properties, thereby providing many niches for microorganisms. Some studies have concluded that soil characteristics have a stronger influence on soil bacterial communities than do other factors such as vegetation type and soil land management (Garbeva et al., 2004; Lauber et al., 2008). Slight changes in soil pH can significantly affect microbial succession in soils (Li et al., 2023), this fact that has been confirmed in tea soils (Xue et al., 2006; Wang et al., 2021). In this study, the bacterial community composition and diversity in tea soils revealed significant variations driven by soil type, plant effects, and their interactions (**Figure 1**). Soil E, with a neutral pH and higher levels of exchangeable cations, showed the highest bacterial richness, and soils A and B with lower pH showed the lowest bacterial richness (**Figure 1A**). These findings suggest that while tea plants are suitable for growing in acidic soil due to nutrient absorption and the accumulation of secondary metabolites of tea leaves, extremely acidic conditions may negatively affect bacterial diversity, which is crucial for maintaining soil health and function (Zeng et al., 2021). In recent years, some tea gardens have responded to the call of green agriculture, organic fertilizer was applied for long term and lead to the gradually increase in soil pH and soil organic matter, which have an impact on bacterial diversity (Ji et al., 2018; Ye et al., 2022). This practice promoted greater bacterial diversity and improved soil fertility, aligning with the growing need for ecological agricultural practices to mitigate soil degradation and acidification in tea-growing regions. Besides, soils with plants exhibited slightly higher richness and different bacterial structure compared to soils without plants across most soil types (**Figure 1B**). This suggested that root exudates and other plant-mediated processes contributed to creating microhabitats for additional bacterial taxa, but these effects were secondary to the dominant influence of soil properties (Hartman and Tringe, 2019).

### 4.2 Effects of different soil types on soil bacterial communities and function

Soil microbial community structure was as an indicator for evaluating soil quality, and analyzing the structure and composition of microbial communities could provide useful information about the complexity of soil ecosystems (Fierer et al., 2021). Tea plants usually cultivated continuously for many years, and this historical background had profound effects on the microbial community structure of the soil, such as the dominant of *Acidobacteria* (Kalam et al., 2020). In a large-scale survey of soils in tea plantations, *Proteobacteria* was identified as the dominant phylum, followed by *Chloroflexi, Bacteroidetes*, and *Acidobacteria*, whereas Ktedonobacteraceae was the dominant family (Kui et al., 2021). However, different compositions of bacterial communities were detected in the present study. *Proteobacteria, Actinobacteriota, Firmicutes*, and *Acidobacteriota* were the dominant phyla and *Acidothermaceae*, *Solirubrobacteraceae*, and *Acetobacteraceae* were the dominant families across all soil types, which indicated their adaptability to diverse soil environments (**Figure 1C**). The correlation analysis between soil chemical properties and bacterial community showed that soil pH was the strongest driver shaping bacterial diversity and composition distribution across different soil types (**Figure 2**). Some studies also reported this opinion in different ecosystems: pH could be a primary factor determining soil bacterial spatial distribution (Rousk et al., 2010; Shen et al., 2013). Soil pH further influenced the solubility of nutrients and the availability of cations (Ca^2+^, Mg^2+^) and create the favorable conditions for specific bacterial taxa such as *Gemmatimonadota*, *Chloroflexi*, *Bacteroidota*, and *Myxococcota*. Other study indicated that the C/N ratio was also the critical drivers of the spatial variations in enzymatic activities and microbial community compositions (Xu et al., 2020). In this study, the bacterial diversity was negatively related to the soil C/N ratio, which indicated that bacteria benefit from soil with a low C/N ratio (Bossuyt et al., 2001). While acidic soils favor specialized and acid-tolerant bacteria such as *Acidothermaceae* and *Solirubrobacteraceae*, an appropriate increase in pH support a more diverse and functionally versatile bacterial community. Optimizing soil pH and the balance of nutrient levels could promote bacterial diversity and enhance soil functions, further improving the soil ecological stability.

Plant–soil microbe interactions are complex, making it challenging to generalize their effects. In some systems, soil microbe could be harmful to plants due to high pathogen prevalence, while in other systems, some beneficial microbes were more influential for plants (Bagchi et al., 2010; Ke and Wan, 2020). In brief, it was obvious that the effect of the presence of plants on soil microbial communities. In our study, the comparative analysis of ASV abundances between soils with and without plants provides valuable insights into the bacterial communities associated with plant effects and their functional roles (**Figure 3**). At the family level, the number of DAAs was significantly higher compared to the phylum level, highlighting the more taxonomic resolution provided by family-level analysis. Soil D exhibited the highest number of DAAs (165), indicating a significant bacterial response to plant effects in this soil type with favorable pH and nutrient status (**Figure 3A**). Previous studies focused on how different soil types drive significantly different bacterial communities in different crops (Ramírez et al., 2020; Xu et al., 2020), and revealed the important role of the soil environment as a fundamental factor for the composition of the bacterial community. While soil type influences bacterial community composition, the highly selective role played by the plants themselves cannot be ignored (Marschner et al., 2004). Our study comprehensively considered the effects of soil type and plant effect and screened the common DAAs to all soil types, further clarifying the core bacterial communities (**Figure 3B**). Among them, *KF-JG30-C25*, *JG36_TzT_191* and *Acidobacteriaceae_subfroup1* showed significantly higher abundance in soil with plants. These findings suggest that certain bacterial taxa may consistently associate with plants across different soil types, forming a core bacterial group that supports plant growth and soil function under varying conditions. Some members of the *Acidobacteriaceae* could support plant growth in oligotrophic soil environments and have capable of degrading complex biopolymers such as xylan, pectin, and chitin (Richards et al., 1997). Another study found that *Acidobacteriaceae* showed positive correlations with *Ktedonobacteraceae* and *Xanthobacteraceae* in the root interior, suggesting their potential role in promoting tea plant growth (Chen et al., 2021). *KF-JG30-C25* was employed to identify the *Acidobacteria Granulicella* sp. and potentially played a role in the metabolism and nutrient cycling of soil organic matter (Costa et al., 2020). By identifying bacterial taxa that consistently respond to plant effects, this study highlighted the potential for developing microbial-based strategies to optimize soil health and plant productivity in other cropping systems. Furthermore, the identification of DAAs provides a foundation for future research aimed at isolating and characterizing these beneficial microbes, potentially leading to their application as biofertilizers or biocontrol agents. Such approaches could help reduce the reliance on chemical inputs, contributing to more sustainable agricultural practices.

### 4.3 Effects of different soil types on tea nutrient status

Plants efficiently absorbed trace elements from the soil solution, whether in free ionic forms or as complexes. and changing the pH of the surrounding root environment can greatly enhance the availability of specific elements (Kabata, 2004). So, the effects of soil properties were identified as the main factor that regulate plant element availability. In a study on soils with a range of pH (3.29, 4.74, and 5.32), the contents of K, Ca, Mg, Mn, P, and S in the tea leaves showed a significant upward trend as the soil pH increased and the soil Al content decreased (Jia et al., 2023). Another study on soils from 20 tea plantations, and on the leaves of the tea plants growing there, showed that an adequate supply of macronutrients and zinc could enhance the contents of polyphenols, FAAs, and caffeine in the leaves (Tseng and Lai, 2022). In this study, the different soil properties affected the nutrient contents of tea new leaves. Soil C, as the conventional management soil, although the pH was suitable, but the nutrient was limited. The growth and quality of tea leaves was obviously limited, but it was conducive the accumulation of catechins. This suggests that under suboptimal nutrient conditions, tea plants may prioritize secondary metabolite production as a stress response to nutrient limitations. Soil D showed the best combined conditions with a moderate pH and good nutrient supply (K, Ca, Mg, S), resulting in improved dry weight and nitrogen and caffeine accumulation of tea new leaves (**Figures 4**, **5**). These findings indicated the importance of maintaining balanced soil nutrient profiles for optimizing both the yield and quality of tea leaves, particularly for metabolites such as nitrogen-containing compounds and caffeine. The enrichment coefficients of N, Mn, C, P, and Mg in tea leaves and quality indexes (tea polyphenols, theanine, and caffeine) showed a significant increasing trend with increasing soil pH (Jia et al., 2024), and the aroma characteristics of tea leaves showed decreasing trends with a decrease in the soil pH (Wang et al., 2023). So, soil pH was shown to play an important role in influencing the essential nutrients of tea leaves. Besides, the second harvest showed lower total nitrogen and catechin contents, but increased FAAs, Ca and Mg contents, which indicated the tea plant preferentially allocated nitrogen for the synthesis of amino acids after successive harvests, while the accumulation of other secondary metabolites was inhibited. Deficiencies in some essential nutrients will affect plant metabolism with subsequent impacts on tea quality. A previous study found that the catabolism of minerals in tea plants influenced the accumulation of amino acids, flavonoids, and glycosides (Huang et al., 2022). Thus, regular monitoring of soil and leaf nutrient contents can guide fertilization practices and develop tailored soil amendments to ensure an adequate and balanced supply of macronutrients and micronutrients.

### 4.4 Relationships among soil properties, bacterial features and tea nutrient status

Soil chemical properties played a critical role in determining the nutrient status of plant, directly influencing not only the plant growth and development but also the synthesis of specialized metabolites (El-Ramady et al., 2014). Among the soil properties, soil pH has been shown to exert significant effects on the metabolic composition of tea leaves. According to Wen et al. (2021), soil pH plays a pivotal role in improving the synthesis of key metabolites including polyphenols, catechins, and amino acids in tea leaves. Soil P, K, and Mg, particularly under biochar application, have demonstrated positive correlations with their corresponding nutrient contents in tea leaves (Yan et al., 2021). In this study, soil chemical properties positively improved the leaf nutrients, particularly N, K, Mg, and Al, and participated in the synthesis of metabolites (catechins and caffeine) (**Figure 6**). On the other hand, soil nutrient deficiencies have been reported to adversely affect the metabolic profile and quality of tea leaves. Zhou et al. (2022) demonstrated that nutrient deficiencies reduced chlorophyll synthesis and decrease the accumulation of several amino acids, leading to declines in tea color, taste, and aroma. This suggests the importance of maintaining balanced soil fertility to sustain high-quality tea production. Proper soil management, including nutrient amendments and pH optimization, is critical for enhancing both primary and secondary metabolism in tea plants. Future research could consider increasing the sample size by adding more replicates to reduce the limitations of statistical results, and further enhance the relationship reliability.

Soil microbial communities, particularly bacterial diversity, play an equally critical role in influencing plant nutrient dynamics and metabolic profiles. Preserving soil bacterial diversity could improve positive plant-soil feedback and thereby plant growth (Weidner et al., 2015). Liu et al. (2021) showed that soil nutrient levels explained most of the variation in annual rice yield, while bacteria diversity indirectly affected rice yield through enzyme activities. But Yi et al. (2022) indicated that increasing soil bacterial richness and diversity could not increase tea yield, and they did not investigate the relationship between bacterial diversity and tea nutrient content. In our study, bacterial diversity was positively correlated with FAAs and TN content in tea new leaves. Besides, soil bacterial communities were positively correlated with N, K, Mg Al FAAs and caffeine content at both harvests (**Figure 6**). It suggests that soil bacterial communities may indirectly promote plant growth and quality formation by regulating nutrient supply and secondary metabolite accumulation. The roles and functions of microbes in plant development have been widely studied. For example, *Bacillus* and *Trichoderma* species have been shown to enhance seed germination rates and seedling vigor in soybeans by improving nutrient acquisition and root development (Bakhshandeh et al., 2020). In tea plant, *Bacillus subtilis* and *Pseudomonas corrugata* were found to have growth promoting property both in tissue culture as well as seed raised plants of tea (Pandey et al., 2000). Specific rhizobacteria such as *Brevibacterium sediminis* A6, isolated from the tea rhizosphere, have demonstrated plant growth-promoting potential (Chopra et al., 2020). Some Bacillus strains (*Paenibacillus* sp. YN15, *Bacillus* sp. BIHB 344, and *Bacillus* sp. DTG11) were also indicated to have tea growth-promoting features such as nitrogen fixation and phosphate solubilization (Bag et al., 2022). Xin et al. (2024) utilized a synthetic microbial community (most from phylum *Proteobacteria*) from tea roots of high-theanine cultivars, which resulted in increasing in the theanine content of tea plants and imparted tolerance to nitrogen deficiency in *Arabidopsis*. Based on these findings, we hypothesize that certain functional bacteria identified in this study may play key roles in regulating nitrogen metabolism and secondary metabolite accumulation. To further confirm the role of bacterial communities in tea metabolism, future studies need to validate these observations through molecular biology and ecological experiments.

## 5 Conclusions

This study indicated the regulatory roles of soil chemical properties and bacterial communities in shaping tea plant growth and quality, emphasizing the complex relationships among soil, bacteria and plants. Adjusting soil pH through regular monitoring and appropriate amendments can directly influence the composition of microbial communities. For instance, soils with lower pH levels required balanced fertilization to prevent excessive acidification while maintaining bacterial activity. Additionally, organic fertilization or microbial inoculants can promote key bacterial taxa associated with improved primary and secondary metabolism in tea plants, meanwhile, avoiding overuse of chemical pesticides and fertilizers, which can harm beneficial microbes, is essential for maintaining soil health and ensuring the long-term sustainability of tea production. In the future, integrate metagenomics and metabolomics approaches to unravel the contributions of key functional microbial communities to tea plant growth and quality formation. These insights will help develop more targeted soil management strategies and optimize the use of microbial inoculants, leading to better tea quality and more sustainable production practices.

## Data Availability

The datasets presented in this study can be found in online repositories. The names of the repository/repositories and accession number(s) can be found in the article/**Supplementary Material**.

## Supplementary material

Supplementary Figure 1 The rarefaction curve for all soil samples.

Supplementary Table 1 The initial properties of different soil types.

Supplementary Table 2 Sequences of primers used for soil bacteria analysis.

Supplementary Table 3 Sequence information after quality filtering and denoising in soil bacteria analysis.

Supplementary Table 4 Mineral contents of mature tea mature leaves and tea roots.
