# Supplementary material for: Soil chemical factors contributing to differences in bacterial communities among tea field soils and their relationships with tea quality
Source: Front Plant Sci. 2025 Jan 30;16:1540659. doi: 10.3389/fpls.2025.1540659 (PMC11821660; doi:10.3389/fpls.2025.1540659)
Supplement: Supplementary Figure 1 — The rarefaction curve for all soil samples. [file DataSheet1.docx]

Supplementary Material

# Supplementary Figures and Tables

## Supplementary Figures


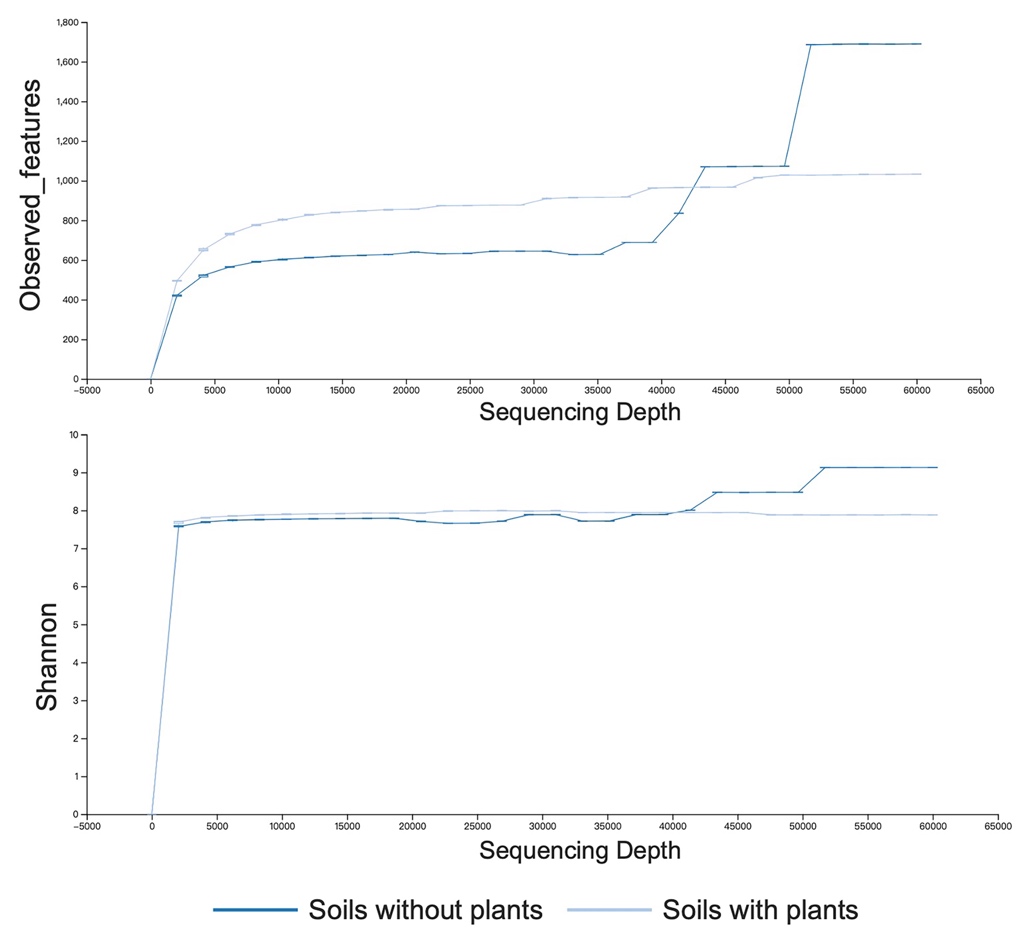


**Supplementary Figure 1.** The Rarefaction curve for all soil samples.

## Supplementary Tables

**Supplementary Table 1** The initial properties of different soil types

| Soil types | pH | EC (ms·cm^-1^) | Total N (%) | Total C (%) |
| --- | --- | --- | --- | --- |
| A | 3.41 | 0.56 | 3.67 | 43.1 |
| B | 3.75 | 0.73 | 2.18 | 25.3 |
| C | 4.16 | 0.19 | 0.49 | 5.07 |
| D | 4.17 | 0.85 | 3.01 | 24.3 |
| E | 5.56 | 0.28 | 1.32 | 13.7 |

**Supplementary Table 2** The sequence details of primers during soil bacteria analysis

| Primer | Sequence | Base in Adapter | Bases for Sample Sheet |
| --- | --- | --- | --- |
| 16S_V4_515F | TCGTCGGCAGCGTCAGATGTGTATAAGAGACAGNNNNNNGTGCCAGCMGCCGCGGTAA | - | - |
| 16S_V4_806R | GTCTCGTGGGCTCGGAGATGTGTATAAGAGACAGNNNNNNGGACTACHVGGGTWTCTAAT | - | - |
| Nextera_i7_N701 | CAAGCAGAAGACGGCATACGAGATTCGCCTTAGTCTCGTGGGCTCGG | TCGCCTTA | TAAGGCGA |
| Nextera_i7_N702 | CAAGCAGAAGACGGCATACGAGATCTAGTACGGTCTCGTGGGCTCGG | CTAGTACG | CGTACTAG |
| Nextera_i7_N703 | CAAGCAGAAGACGGCATACGAGATTTCTGCCTGTCTCGTGGGCTCGG | TTCTGCCT | AGGCAGAA |
| Nextera_i7_N704 | CAAGCAGAAGACGGCATACGAGATGCTCAGGAGTCTCGTGGGCTCGG | GCTCAGGA | TCCTGAGC |
| Nextera_i7_N705 | CAAGCAGAAGACGGCATACGAGATAGGAGTCCGTCTCGTGGGCTCGG | AGGAGTCC | GGACTCCT |
| Nextera_i7_N706 | CAAGCAGAAGACGGCATACGAGATCATGCCTAGTCTCGTGGGCTCGG | CATGCCTA | TAGGCATG |
| Nextera_i7_N707 | CAAGCAGAAGACGGCATACGAGATGTAGAGAGGTCTCGTGGGCTCGG | GTAGAGAG | CTCTCTAC |
| Nextera_i7_N710 | CAAGCAGAAGACGGCATACGAGATCAGCCTCGGTCTCGTGGGCTCGG | CAGCCTCG | CGAGGCTG |
| Nextera_i7_N711 | CAAGCAGAAGACGGCATACGAGATTGCCTCTTGTCTCGTGGGCTCGG | TGCCTCTT | AAGAGGCA |
| Nextera_i7_N712 | CAAGCAGAAGACGGCATACGAGATTCCTCTACGTCTCGTGGGCTCGG | TCCTCTAC | GTAGAGGA |
| Nextera_i7_N714 | CAAGCAGAAGACGGCATACGAGATTCATGAGCGTCTCGTGGGCTCGG | TCATGAGC | GCTCATGA |
| Nextera_i7_N715 | CAAGCAGAAGACGGCATACGAGATCCTGAGATGTCTCGTGGGCTCGG | CCTGAGAT | ATCTCAGG |
| Nextera_i5_S502 | AATGATACGGCGACCACCGAGATCTACACCTCTCTATTCGTCGGCAGCGTC | CTCTCTAT | CTCTCTAT |
| Nextera_i5_S503 | AATGATACGGCGACCACCGAGATCTACACTATCCTCTTCGTCGGCAGCGTC | TATCCTCT | TATCCTCT |
| Nextera_i5_S505 | AATGATACGGCGACCACCGAGATCTACACGTAAGGAGTCGTCGGCAGCGTC | GTAAGGAG | GTAAGGAG |
| Nextera_i5_S506 | AATGATACGGCGACCACCGAGATCTACACACTGCATATCGTCGGCAGCGTC | ACTGCATA | ACTGCATA |
| Nextera_i5_S507 | AATGATACGGCGACCACCGAGATCTACACAAGGAGTATCGTCGGCAGCGTC | AAGGAGTA | AAGGAGTA |
| Nextera_i5_S508 | AATGATACGGCGACCACCGAGATCTACACCTAAGCCTTCGTCGGCAGCGTC | CTAAGCCT | CTAAGCCT |
| Nextera_i5_S510 | AATGATACGGCGACCACCGAGATCTACACCGTCTAATTCGTCGGCAGCGTC | CGTCTAAT | CGTCTAAT |
| Nextera_i5_S511 | AATGATACGGCGACCACCGAGATCTACACTCTCTCCGTCGTCGGCAGCGTC | TCTCTCCG | TCTCTCCG |

**Supplementary Table 3** The initial properties of different soil types

| Treatment | input | filtered | percentage of input passed filter | denoised | percentage of input denoised | merged | percentage of input merged | non-chimeric | percentage of input non-chimeric |
| --- | --- | --- | --- | --- | --- | --- | --- | --- | --- |
| A_1 | 34567 | 31339 | 90.66 | 30207 | 87.39 | 28148 | 81.43 | 27910 | 80.74 |
| A_2 | 26684 | 24297 | 91.05 | 23229 | 87.05 | 21075 | 78.98 | 20821 | 78.03 |
| A_3 | 112862 | 101361 | 89.81 | 99161 | 87.86 | 94724 | 83.93 | 92780 | 82.21 |
| A_4 | 59713 | 54332 | 90.99 | 52457 | 87.85 | 48994 | 82.05 | 47844 | 80.12 |
| A_5 | 57593 | 52507 | 91.17 | 50806 | 88.22 | 47858 | 83.1 | 46999 | 81.61 |
| A_without_plants_1 | 61473 | 54989 | 89.45 | 54038 | 87.91 | 51628 | 83.98 | 50535 | 82.21 |
| A_without_plants_2 | 30018 | 27196 | 90.6 | 26323 | 87.69 | 24364 | 81.16 | 23855 | 79.47 |
| A_without_plants_3 | 47118 | 42668 | 90.56 | 41508 | 88.09 | 38805 | 82.36 | 38194 | 81.06 |
| A_without_plants_4 | 32418 | 29476 | 90.92 | 28480 | 87.85 | 26473 | 81.66 | 26059 | 80.38 |
| B_1 | 89212 | 81537 | 91.4 | 79846 | 89.50 | 75371 | 84.49 | 72940 | 81.76 |
| B_2 | 44434 | 40445 | 91.02 | 39192 | 88.20 | 36828 | 82.88 | 36000 | 81.02 |
| B_3 | 39721 | 33433 | 84.17 | 32330 | 81.39 | 29578 | 74.46 | 28972 | 72.94 |
| B_4 | 39244 | 34524 | 87.97 | 33545 | 85.48 | 31619 | 80.57 | 31091 | 79.22 |
| B_without_plants_1 | 48970 | 44146 | 90.15 | 43301 | 88.42 | 41900 | 85.56 | 41675 | 85.1 |
| B_without_plants_2 | 44235 | 39680 | 89.7 | 38794 | 87.70 | 37314 | 84.35 | 36777 | 83.14 |
| B_without_plants_3 | 43949 | 39890 | 90.76 | 39082 | 88.93 | 37638 | 85.64 | 37173 | 84.58 |
| B_without_plants_4 | 37041 | 32063 | 86.56 | 31064 | 83.86 | 28860 | 77.91 | 28574 | 77.14 |
| B_without_plants_5 | 49484 | 44484 | 89.9 | 43259 | 87.42 | 40992 | 82.84 | 39897 | 80.63 |
| E_1 | 49258 | 43262 | 87.83 | 40585 | 82.39 | 35588 | 72.25 | 35352 | 71.77 |
| C_1 | 47406 | 43110 | 90.94 | 41921 | 88.43 | 39609 | 83.55 | 38865 | 81.98 |
| C_2 | 98534 | 89517 | 90.85 | 87710 | 89.01 | 83824 | 85.07 | 81691 | 82.91 |
| C_3 | 108452 | 98105 | 90.46 | 96568 | 89.04 | 92946 | 85.7 | 90506 | 83.45 |
| C_4 | 80812 | 73288 | 90.69 | 71979 | 89.07 | 69043 | 85.44 | 67632 | 83.69 |
| C_5 | 100580 | 91355 | 90.83 | 89900 | 89.38 | 85436 | 84.94 | 82985 | 82.51 |
| C_without_plants_1 | 47405 | 42922 | 90.54 | 41940 | 88.47 | 39792 | 83.94 | 39497 | 83.32 |
| C_without_plants_2 | 45652 | 41563 | 91.04 | 40676 | 89.10 | 37577 | 82.31 | 36633 | 80.24 |
| C_without_plants_3 | 46130 | 41250 | 89.42 | 40156 | 87.05 | 37979 | 82.33 | 37689 | 81.7 |
| C_without_plants_4 | 43984 | 39653 | 90.15 | 38798 | 88.21 | 37330 | 84.87 | 36842 | 83.76 |
| D_1 | 47989 | 43328 | 90.29 | 41771 | 87.04 | 39227 | 81.74 | 38869 | 81 |
| D_2 | 79045 | 71965 | 91.04 | 70225 | 88.84 | 66663 | 84.34 | 65897 | 83.37 |
| D_3 | 36049 | 32656 | 90.59 | 31455 | 87.26 | 29552 | 81.98 | 29249 | 81.14 |
| D_4 | 40723 | 36906 | 90.63 | 35515 | 87.21 | 33312 | 81.8 | 32993 | 81.02 |
| D_without_plants_1 | 78851 | 70614 | 89.55 | 68242 | 86.55 | 63357 | 80.35 | 62126 | 78.79 |
| D_without_plants_2 | 31388 | 28370 | 90.38 | 26892 | 85.68 | 24415 | 77.78 | 24013 | 76.5 |
| D_without_plants_3 | 26648 | 24120 | 90.51 | 22937 | 86.07 | 20729 | 77.79 | 20476 | 76.84 |
| D_without_plants_4 | 35953 | 32436 | 90.22 | 31183 | 86.73 | 28645 | 79.67 | 28057 | 78.04 |
| E_2 | 38981 | 34884 | 89.49 | 32097 | 82.34 | 27819 | 71.37 | 27714 | 71.1 |
| E_3 | 102256 | 90896 | 88.89 | 86807 | 84.89 | 79313 | 77.56 | 78600 | 76.87 |
| E_4 | 40735 | 36602 | 89.85 | 34880 | 85.63 | 32466 | 79.7 | 32230 | 79.12 |
| E_without_plants_1 | 103163 | 92554 | 89.72 | 88898 | 86.17 | 81862 | 79.35 | 80228 | 77.77 |
| E_without_plants_2 | 32696 | 28801 | 88.09 | 26474 | 80.97 | 22630 | 69.21 | 22523 | 68.89 |
| E_without_plants_3 | 44326 | 40003 | 90.25 | 37560 | 84.74 | 33416 | 75.39 | 33063 | 74.59 |
| E_without_plants_4 | 52369 | 47056 | 89.85 | 44501 | 84.98 | 40444 | 77.23 | 39928 | 76.24 |
| E_without_plants_5 | 45547 | 40016 | 87.86 | 37316 | 81.93 | 32630 | 71.64 | 32296 | 70.91 |

**Supplementary Table 3** Mineral contents of mature tea leaves and tea roots.

|  | Soil  type | Al  (mg g^-1^ DW) | Fe  (mg g^-1^ DW) | Na  (mg g^-1^ DW) | B  (mg g^-1^ DW) | P  (mg g^-1^ DW) | S  (mg g^-1^ DW) | Ca  (mg g^-1^ DW) | Cu  (mg g^-1^ DW) | K  (mg g^-1^ DW) | Mg  (mg g^-1^ DW) | Mn  (mg g^-1^ DW) | Zn  (mg g^-1^ DW) |
| --- | --- | --- | --- | --- | --- | --- | --- | --- | --- | --- | --- | --- | --- |
| ML | A | 1.34±0.11b | 0.10±0.01c | 0.09±0.02b | 0.03±0.00bc | 1.35±0.07cd | 1.60±0.05c | 7.80±0.74c | 0.004±0.001bc | 1.94±0.34c | 1.93±0.31d | 2.19±0.19a | 0.02±0.00a |
|  | B | 1.35±0.31b | 0.12±0.02bc | 0.13±0.04ab | 0.04±0.00b | 4.39±0.39a | 1.88±0.09b | 10.80±1.59b | 0.003±0.000c | 5.69±0.59a | 2.63±0.13c | 1.81±0.37a | 0.01±0.00a |
|  | C | 2.64±0.37a | 0.34±0.09a | 0.13±0.02ab | 0.04±0.00a | 1.88±0.63c | 1.38±0.11d | 8.35±0.68c | 0.006±0.001a | 3.42±0.59b | 2.13±0.22d | 2.22±0.25a | 0.01±0.00a |
|  | D | 1.29±0.18b | 0.15±0.03bc | 0.15±0.03a | 0.03±0.00c | 2.83±0.07b | 2.27±0.18a | 12.91±0.68a | 0.005±0.001ab | 5.93±1.04a | 4.36±0.07a | 2.02±0.14a | 0.01±0.00a |
|  | E | 0.66±0.32c | 0.21±0.02b | 0.11±0.04ab | 0.02±0.00d | 1.05±0.08d | 1.58±0.09cd | 14.29±0.91a | 0.003±0.000c | 3.19±0.30b | 3.53±0.22b | 0.40±0.17b | 0.01±0.00a |
| Root | A | 0.70±0.08b | 0.31±0.04c | 0.41±0.06ab | 0.01±0.00c | 3.54±0.67c | 2.45±0.20b | 1.40±0.08d | 0.02±0.01b | 6.05±0.57b | 1.95±0.21d | 0.15±0.04b | 0.10±0.01c |
|  | B | 0.77±0.05b | 0.48±0.03c | 0.27±0.02cd | 0.01±0.00bc | 9.10±1.00a | 2.68±0.21b | 1.94±0.22c | 0.01±0.00b | 10.13±1.57a | 3.88±0.29c | 0.18±0.04b | 0.22±0.03b |
|  | C | 2.76±0.38a | 1.57±0.40b | 0.42±0.07a | 0.01±0.00b | 5.49±0.60b | 2.81±0.27ab | 1.22±0.04d | 0.03±0.00a | 8.70±0.75a | 3.05±0.27cd | 0.21±0.02b | 0.12±0.02c |
|  | D | 0.78±0.10b | 0.56±0.13c | 0.31±0.08bc | 0.01±0.00b | 9.32±1.68a | 2.66±0.12b | 3.08±0.27b | 0.01±0.00b | 10.35±2.55a | 6.24±0.57b | 0.35±0.08a | 0.27±0.03a |
|  | E | 2.24±1.15a | 2.89±1.08a | 0.15±0.01d | 0.01±0.00a | 2.05±0.61c | 3.14±0.17a | 3.99±0.55a | 0.01±0.00b | 9.29±0.55a | 8.20±1.32a | 0.04±0.01c | 0.03±0.00d |

*Values are mean ± SD (n = 5). According to Turkey’s test, different letters indicate that means are significantly different (P < 0.05）

ML: Mature Leaves
